# Analytical Modeling on Investigating the Electric Field Shift Behavior of GaN Vertical FinFETs

**DOI:** 10.1021/acsomega.6c03756

**Published:** 2026-06-11

**Authors:** Ancy Michel, Mohd Syamsul, T. S. Arun Samuel, I. Vivek Anand, Navin M. George, D. Nirmal

**Affiliations:** † Department of Electronics and Communication Engineering, 121735 Karunya Institute of Technology and Sciences, Coimbatore 641114, Tamil Nadu, India; ‡ Institute of Nano Optoelectronics Research and Technology (INOR), 26689 Universiti Sains Malaysia, Bayan Lepas, Pulau Pinang 11900, Malaysia; § Faculty of Science and Engineering, Waseda University, Tokyo 169-8555, Japan; ∥ Department of Electronics and Communication Engineering, 267718 National Engineering College, Kovilpatti 628503, Tamil Nadu, India; ⊥ Department of Electronics and Communication Engineering, 243659 Nehru Institute of Engineering and Technology, Coimbatore 641105, Tamil Nadu, India

## Abstract

GaN vertical FinFETs have emerged as an increasingly prevalent semiconductor technology for RF and power applications. To fully exploit GaN power transistors, accurate and reliable physics-driven models have been required. An analytical model based on surface potential for vertical GaN FinFETs featuring a submicron fin channel on a GaN substrate is presented, considering the electrostatic influence of a field plate gate. A two-dimensional Poisson equation is analyzed using the parabolic estimation to characterize the electrostatic potential along the fin channel. Thereby, the surface potential model provides insight into the determination of threshold voltage, electrostatic control, and drain current. In addition, an ON-resistance model has also been derived to analyze the device performance. Then, the derived models are compared with the simulated results of a field plate-added stepped gate device obtained from TCAD. The simulated device based on this analytical model provides an ON current of 4 kA/cm^2^ associated with its specific ON resistance of 0.012 mΩ·cm^2^. This analytical approach provides a reliable platform for optimizing the electrostatic design of GaN vertical FinFET devices.

## Graphical abstract

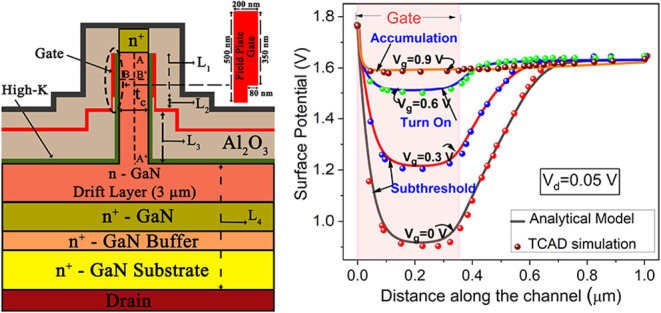

## 1 Introduction

Gallium nitride (GaN) is evolving as a substantial contributor to the current era of electronics, which is being influenced by the developments in semiconductor technology. GaN is notable for its outstanding features, including a broad band gap, high breakdown voltage, and high efficiency at high frequencies, as industries want faster, smaller, and more efficient devices.^1–3^ In light of these attributes, GaN is a frontline material that has the capacity to exceed conventional silicon across various applications, reshaping power electronics and RF systems.^4^ The power electronics applications necessitate higher breakdown voltage and lower ON resistance (*R*_ON_) for handling large current with higher efficiency.^5^ Lateral GaN devices, particularly HEMTs (high electron mobility transistors), are initially dominant in the scenario.^6,7^ However, the normally ON behavior challenges the safety in high-power devices, prompting the adoption of gate recessing and cascode configurations for obtaining a normally OFF nature, making it bulky, which makes a transition shift to vertical architectures.^8^

Vertical GaN devices utilize the perpendicular current flow through the bulk substrate, enabling higher breakdown voltages and a reduced footprint.^9–11^ Several vertical architectures have been demonstrated; among them, a vertical fin field effect transistor (FinFET) does not necessitate epitaxial regrowth and needs only n-homogeneous layers.^12,13^ Furthermore, the excellent field effect control by the fin structure over the channel improves the performance further.^14,15^ Although the quasi-vertical architecture has a cost advantage due to its implementation on a foreign substrate, the higher dislocation density results in lower performance.^16^ Nevertheless, contrasted with the quasi-structure, the device with the same GaN substrate reveals a beneficial effect on field spreading.^17,18^ The GaN vertical FinFET demonstrated a 1200 V breakdown with 2 mΩ·cm^2^ resistance,^19^ and an unprecedented switching performance is displayed by an expanded device area 1200 V, 5 A vertical structured FinFET out of all GaN power devices of the same range.^20^ Gallium oxide (Ga_2_O_3_) vertical FinFETs with a 600 A/cm^2^ drain current density and a 1.6 kV breakdown voltage are demonstrated.^21^ Yet, in comparison with GaN vertical structures, the Ga_2_O_3_ structure is unable to satisfy the fast-switching requirements and reliable thermal management.^22^

The benefits of vertical architecture are not fully exploited because of the lack of complete physical model exposure. The analytical model aids fast evaluation of device electrostatics and leads to easier device optimization steps.^23^ Only a few analytical models for vertical GaN FinFETs have been developed so far. The extraction of ON resistance and channel mobility in vertical FinFETs is reported.^24^ The surface potential modeling and fringing capacitance are reported for gallium oxide fully vertical structures.^25^ The electrostatic behavior at a high bias is modeled, and its dependence on the maximum source-to-drain potential barrier is reported.^26^

While being significantly pertinent to GaN FinFETs, the analytical models describing the field redistribution and gate-to-drift coupling introduced by field plates are still lacking. This work contributes to the mathematical modeling of a GaN vertical power FinFET having a field plate-added gate by formulating appropriate boundary constraints and assumptions. The influence of a field-plated gate on surface potential, electric field across the channel, and ON-resistance models is captured by partitioning the device into distinct vertical regions. The linking of carrier concentration and mobility to the gate-controlled surface potential enables accurate prediction of the conduction behavior of the device. The analytical model is then validated with the experimental device, and the coefficient of determination (*R* ^2^) and root-mean-square error (RMSE) are extracted.

## 2 Device Structure

In the device structure, homogeneous gallium nitride layers comprise a 350 nm n^+^ source region, a 1000 nm n^+^ fin channel, and a 3000 nm drift layer, all stacked on an n^+^ gallium nitride substrate (Figure 1). The substrate serves as the drain region. The high-k hafnium oxide (HfO_2_) is used as a gate oxide, which is deposited between the tungsten gate and the semiconductor to provide effective electrical isolation between them. A spacer oxide such as aluminum oxide (Al_2_O_3_) is deposited on the upper and lower sections of the gates to reduce peak electric fields at gate corners. Besides the experimental structure,^14^ gate length (*L*_g_) is reduced to 350 nm with an 80 nm thickness, and a gate field plate of 120 nm thickness is added along with the gate, designed to enhance DC performance significantly. The drift layer is doped with an electron concentration comparable to that of the fin channel to minimize the voltage drop. The gate dielectric employed in the device structure is hafnium oxide (HfO_2_), with a dielectric strength of 22 and conduction and valence band offsets of approximately 1.5 and 2.9 eV, respectively. The conduction band offset to GaN is 2.1 eV, and that of the valence band offset is 1.4 eV. The interface charge density at the GaN/HfO_2_ interface is present in the range of 10^12^–10^13^ cm^–2^·eV^–1^, and the fixed oxide charge is in the order of 10^12^–10^13^ cm^–2^. To facilitate precise computations and modeling of the structure, the device is segmented into four primary regions, namely, L_1_, L_2_, L_3_, and L_4_, spanning from the source to the drain. Region L_1_ corresponds to the fin channel of 350 nm from the source area, L_2_, the fin channel from 350 to 500 nm, L_3_, the fin channel without gate area, and L_4_, the drain depletion regime. Table 1 provides the parameters employed in the device modeling.

**Figure 1:**
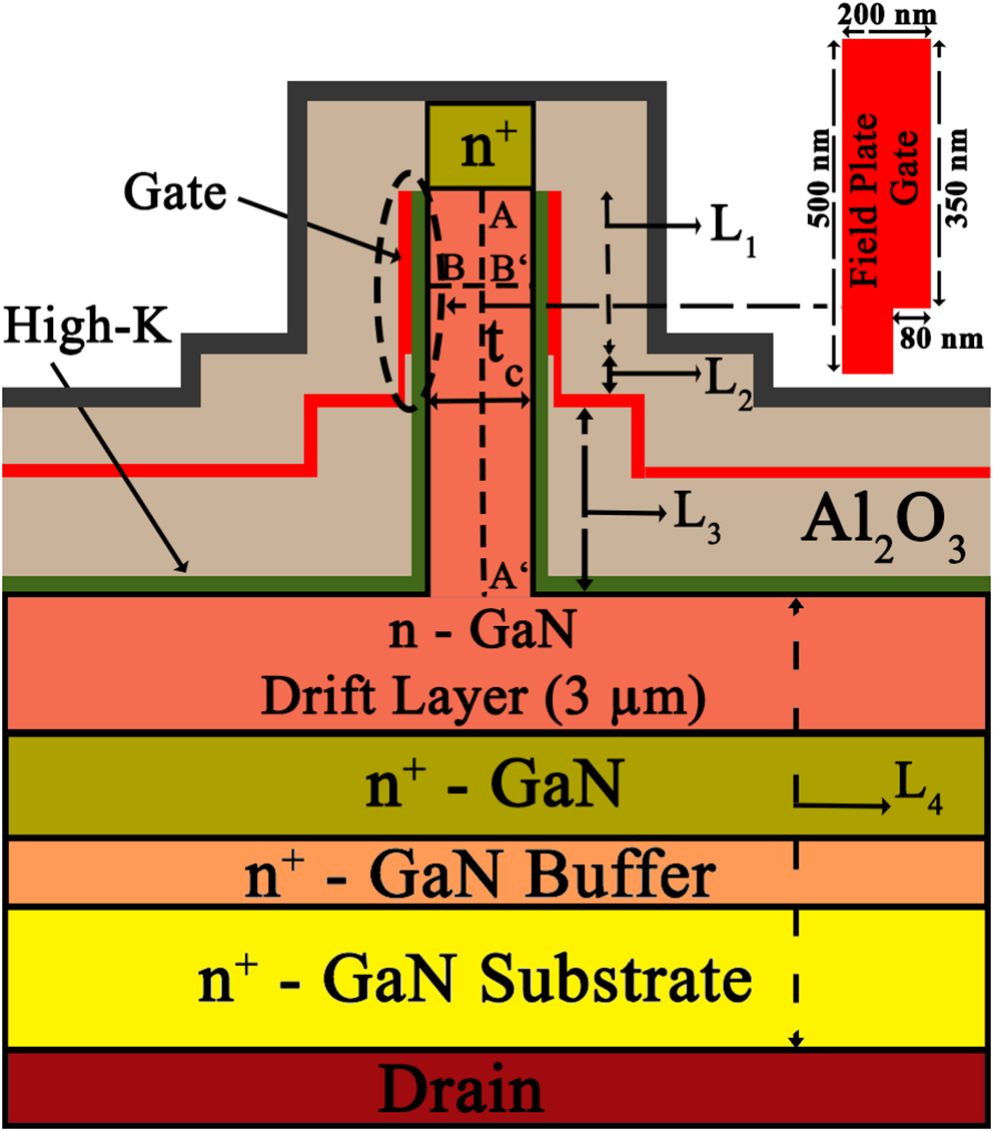
2D schematic view of a step-gated GaN vertical FinFET.

**Table 1:** Parameters Employed in the Device Simulation

notation	parameter	value
*t*_c_	fin channel thickness	0.1 μm
*H*	height of fin	1 μm
*L*_g_	length of the gate	0.35 μm
*W*_d_	thickness of the drift layer	3 μm
*t*_HfO_2__	thickness of HfO_2_	10 nm
φ_m_	work function of tungsten gate	4.85 eV
ε_ox_	permittivity of gate oxide	20 ε_0_
ε_GaN_	permittivity of GaN	8.9 ε_0_
*E*_g_	band gap of GaN	3.4 eV
*N*_d,fin_	doping of the fin channel	4 × 10^16^ cm^–3^
*N*_d,drift_	doping of the drift layer	4 × 10^16^ cm^–3^
*N*_d,source/drain_	doping of the source/drain	1 × 10^19^ cm^–3^

## 3 Analytical Modeling

### 3.1 Surface Potential Modeling

The surface potential distribution in the GaN vertical FinFET is derived from the two-dimensional (2D) Poisson equation and can be represented as

1 $$\frac{\partial^{2}\phi_{i}\left(x,y\right)}{\partial x^{2}}+\frac{\partial^{2}\phi_{i}\left(x,y\right)}{\partial y^{2}}=\frac{qN_{\mathrm{Ri}}}{\varepsilon_{\mathrm{GaN}}}$$

where, ϕ_*i*_(*x*, *y*) represents the surface potential in the *i^th^* region, *N*_*Ri*_ is the dopant concentration in the *i^th^* region, *ε*_GaN_ corresponds to the permittivity of GaN, and *q* corresponds to the electron charge. The second-order (quadratic) polynomial in eq 1 can be resolved by applying a parabolic approximation, represented as

2 $$\phi \left(x,y\right)=a_{0}\left(x\right)+a_{1}\left(x\right)y+a_{2}\left(x\right)y^{2}+a_{3}\left(x\right)y^{3}$$

where, *a*_0_, *a*_1_, *a*_2_, and *a*_3_ are the polynomial coefficients. The boundary conditions can be determined by ensuring the continuity of both the field potential and electric displacement vectors at the interfaces between regions 1, 2, and 3.

(1) Assuming a symmetric channel, the potential at the junction of GaN and gate oxide reflects the surface potential ϕ_*i*_(*x*), which is formulated as
3) $$\phi_{i}\left(x,0\right)=\phi_{i}\left(x,t_{c}\right)=\phi_{i}\left(x\right)$$
  where *t*_c_ represents the thickness of the channel.
(2) The displacement vector of the electric field remains uniform across the interface between the semiconductor and the oxide and is given by
4) $${\frac{\partial \phi_{i}\left(x,y\right)}{\partial y}|}_{y=0}=\frac{-C_{\mathrm{ox}}\left(V_{\mathrm{gi}}-\phi_{i}\left(x\right)\right)}{\varepsilon_{\mathrm{GaN}}}$$
  where $C_{\mathrm{ox}}=\frac{\varepsilon_{{\mathrm{HfO}}_{2}}}{t_{\mathrm{ox}}}$ , *V*_gi_ is the gate voltage, ε_HfO_2__ represents the relative permittivity of HfO_2_, and *t*_ox_ is the gate oxide thickness.
(3) The electric field should diminish to zero at $y=\frac{t_{c}}{2}$ as the channel is symmetric.
5) $${\frac{\partial \phi_{i}\left(x,y\right)}{\partial y}|}_{y=t_{c}/2}=0$$
  Using the given conditions and neglecting higher-order terms of eq 2 result in
6) $$\phi \left(x,y\right)=a_{0}\left(x\right)+a_{1}\left(x\right)y+a_{2}\left(x\right)y^{2}$$

The cubic term in eq 2 is neglected as its value is small compared within the channel approximation. Therefore, it has minimal effect on the surface potential distribution. Then, applying the first boundary condition and taking the derivative of the given equation give

7 $$\frac{\partial \phi \left(x,y\right)}{\partial y}=a_{1}\left(x\right)+2a_{2}\left(x\right)y$$

Applying the boundary condition and equating eq 4 with the central potential, a_1_ (x) is determined as follows:

8 $$a_{1}\left(x\right)=\frac{-C_{\mathrm{ox}}\left(V_{g_{i}}-\phi_{i}\left(x\right)\right)}{\varepsilon_{\mathrm{GaN}}}$$

Substituting *a*_1_(*x*) in eq 7 and *a*_2_(*x*) from eq 2 as follows:

9 $$a_{2}\left(x\right)=\frac{C_{\mathrm{ox}}\left(V_{g_{i}}-\phi_{i}\left(x\right)\right)}{t_{c}\varepsilon_{\mathrm{GaN}}}$$

Substituting eqs 8 and 9 in eq 2 gives

10 $$\phi \left(x,y\right)=\phi_{i}\left(x\right)-\frac{C_{\mathrm{ox}}\left(V_{g_{i}}-\phi_{i}\left(x\right)\right)}{\varepsilon_{\mathrm{GaN}}}y+\frac{C_{\mathrm{ox}}\left(V_{g_{i}}-\phi_{i}\left(x\right)\right)}{t_{c}\varepsilon_{\mathrm{GaN}}}y^{2}$$

11 $$\frac{\partial^{2}\phi_{i}\left(x\right)}{\partial x^{2}}+\frac{C_{\mathrm{ox}}}{\varepsilon_{\mathrm{GaN}}}\frac{\partial^{2}\phi_{i}\left(x\right)}{\partial x^{2}}\frac{t_{c}}{2}-\left(\frac{C_{\mathrm{ox}}}{\varepsilon_{\mathrm{GaN}}t_{c}}\right)\frac{\partial^{2}\phi_{i}\left(x\right)}{\partial x^{2}}\frac{t_{c}^{2}}{4}+\frac{2C_{\mathrm{ox}}\left(V_{g_{i}}-\phi_{i}\left(x\right)\right)}{t_{c}\varepsilon_{\mathrm{GaN}}}=\frac{qN_{R_{i}}}{\varepsilon_{\mathrm{GaN}}}$$

Using *K*_*i*_ and β_*i*_, the differential equation with exciting force can be solved, and the complete solution for ϕ_*i*_(*x*) is given by

12 $$\phi_{i}\left(x\right)=P_{i}\exp\left(K_{i}z\right)+Q_{i}\exp\left(-K_{i}z\right)-\frac{\beta_{i}}{K_{i}^{2}}$$

$$K_{i}^{2}=\frac{2C_{\mathrm{ox}}}{t_{c}\varepsilon_{\mathrm{GaN}}}\,\;\mathrm{and}{\;\;\beta }_{i}=\frac{qN_{R_{i}}}{\varepsilon_{\mathrm{GaN}}}-K_{i}^{2}V_{g_{i}}$$ 13

For *i* = 1 (*L*_g_ of 350 nm), surface potential is obtained as

14a $$\phi_{1}\left(x\right)=P_{1}\exp\left(K_{1}z\right)+Q_{1}\exp\left(-K_{1}z\right)-\frac{\beta_{1}}{K_{1}^{2}}$$

The value of *C*_ox_ for region 1 is given as

14b $$C_{\mathrm{ox}}=\frac{\varepsilon_{{\mathrm{HfO}}_{2}}}{t_{{\mathrm{HfO}}_{2}}}$$

For *i* = 2, where the field plate is present, considering both oxides, the surface potential is given by

15a $$\phi_{2}\left(x\right)=P_{2}\exp\left(K_{2}\left(x-L_{1}\right)\right)+Q_{2}\exp\left(-K_{2}\left(x-L_{1}\right)\right)-\frac{\beta_{2}}{K_{2}^{2}}$$

15b $$C_{\mathrm{ox}}=\frac{\varepsilon_{\mathrm{ox}}}{t_{\mathrm{eq}}}$$

where *t*_eq_ (equivalent oxide thickness) can be explained as

15c $$t_{\mathrm{eq}}=\frac{\varepsilon_{\mathrm{ox}}t_{{\mathrm{HfO}}_{2}}}{\varepsilon_{{\mathrm{HfO}}_{2}}}$$

For *i* = 3, the surface potential of the channel area without a gate can be defined as

16a $$\phi_{3}\left(x\right)=P_{3}\exp\left(K_{3}\left(x-L_{1}-L_{2}\right)\right)+Q_{3}\exp\left(-K_{3}\left(x-L_{1}-L_{2}\right)\right)-\frac{\beta_{3}}{K_{3}^{2}}$$

Here,

16b $$C_{\mathrm{ox}}=\frac{\varepsilon_{\mathrm{ox}}}{t_{\mathrm{eq}}}$$

where *t*_eq_ represents the equivalent oxide thickness.

16c $$t_{\mathrm{eq}}=t_{\mathrm{ox}}+\frac{\varepsilon_{\mathrm{ox}}t_{{\mathrm{HfO}}_{2}}}{\varepsilon_{{\mathrm{HfO}}_{2}}}$$

For *i* = 4,

17 $$\phi_{4}\left(x\right)=P_{4}\exp\left(K_{4}\left(x-L_{1}-L_{2}-L_{3}\right)\right)+Q_{4}\exp\left(-K_{4}\left(x-L_{1}-L_{2}-L_{3}\right)\right)-\frac{\beta_{4}}{K_{4}^{2}}$$

The work function of the semiconductor (GaN) aligned in a metal–semiconductor interface region is dependent on the electron affinity of the semiconductor, energy in the mid band gap, and the fermi level offset.

18 $$\phi_{\mathrm{GaN}}=\chi_{\mathrm{GaN}}+\frac{E_{g}}{2q}+\phi_{\mathrm{Fi}}$$

where

19 $$\phi_{\mathrm{Fi}}=V_{t}\ln\left(\frac{N_{R}}{n_{i}}\right)$$

Boundary condition 1: At the source end, the surface charge potential can be obtained by

20 $$\phi_{s1}\left(0\right)=-\frac{K_{B}T}{q}\ln\left(\frac{N_{s}}{n_{i}}\right)=V_{\mathrm{bi}}$$

Boundary condition 2: At the interface of the source and channel, the surface charge potential can be obtained by

21 $$\phi_{s1}={\phi_{s2}|}_{x=L_{1}},\mathrm{and},\frac{\partial \phi_{s1}}{\partial x}={\frac{\partial \phi_{s2}}{\partial x}|}_{x=L_{1}}$$

Boundary condition 3: The surface charge potential at the channel–drain junction can be determined by

22 $$\phi_{s2}={\phi_{s3}|}_{x=L_{1}+L_{2}},\mathrm{and},\frac{\partial \phi_{s2}}{\partial x}={\frac{\partial \phi_{s3}}{\partial x}|}_{x=L_{1}+L_{2}}$$

Boundary condition 4: At the drain depletion end, the surface charge potential can be obtained by

23 $$\phi_{s3}=\frac{K_{B}T}{q}\ln\left(\frac{N_{d}}{n_{i}}\right)+V_{d}=V_{\mathrm{bi}}+V_{d}$$

By setting ϕ_s3_ as the sum of the built-in potential and the applied drain bias, the model assumes that the potential in region 4 is constant. Therefore, the boundary condition for region 4 is the same as that of region 3. Applying the boundary condition in eq 16 results in

$$P_{1}\exp\left(K_{1}L_{1}\right)+Q_{1}\exp\left(-K_{1}L_{1}\right)-\frac{\beta_{1}}{K_{1}^{2}}-\frac{\beta_{2}}{K_{2}^{2}}=P_{2}+Q_{2}$$ 24

In general, P_i_ can be expressed as

25 $$P_{i}=0.5\left\{P_{i-1}\left(1+\frac{K_{i-1}}{K_{i}}\right)\exp\left[-\left(K_{i}-K_{i-1}\right)\right]\sum_{j=0}^{i-2}\left(i-2\right)L_{j}+Q_{i-1}\left(1-\frac{K_{i-1}}{K_{i}}\right)\exp\left[-\left(K_{i}+K_{i-1}\right)\right]\sum_{j=0}^{i-2}\left(i-2\right)L_{j}-\frac{\beta_{i-1}}{K_{i-1}^{2}}\exp\left(-K_{i}\right)\left(\sum_{j=0}^{i-2}\left(i-2\right)L_{j}+\Gamma \right)\right\}+\frac{\beta_{i}}{K_{i}^{2}}$$

Similarly,

26 $$Q_{i}=0.5\left\{P_{i-1}\left(1-\frac{K_{i-1}}{K_{i}}\right)\exp\left(K_{i}+K_{i-1}\right)\sum_{j=0}^{i-2}\left(i-2\right)L_{j}-\frac{\beta_{i-1}}{K_{i-1}^{2}}\exp\left(-K_{i}\right)\left(\sum_{j=0}^{i-2}\left(i-2\right)L_{j}+\Gamma \right)\right\}+\frac{\beta_{i}}{K_{i}^{2}}$$

where Γ is a fitting value that ranges from −0.07 to 0.07 and is used to compare the mathematical data of the surface potential with the simulation findings. The electric field (EF) along the lateral and vertical directions can be determined as

27 $$E_{\mathrm{xi}}=-\frac{\partial \phi_{i}\left(x,y\right)}{\partial x},\mathrm{and},E_{\mathrm{yi}}=-\frac{\partial \phi_{i}\left(x,y\right)}{\partial y}$$

### 3.2 ON Resistance

The total drain current (*I*_d_) in the vertical structure is the contribution of both drift and diffusion currents on either side of the symmetric device, which can be expressed as

28 $$I_{d}=2\left(I_{\mathrm{drift}}+I_{\mathrm{diffusion}}\right)$$

We consider that the channel resistance *R*_ch_ comprises a parallel configuration of *R*_MOS_ and *R*_fin_. *R*_s_ is the total resistance, which is not biased by the gate and is determined as

29 $$R_{S}=R_{\mathrm{SC}}+R_{n}+R_{\mathrm{spread}}+R_{\mathrm{drift}}+R_{\mathrm{sub}}+R_{\mathrm{DC}}$$

where *R*_SC_ is the resistance obtained from the source contact, *R*_n_ is the resistance in the source region, *R*_spread_ + *R*_drift_ is the resistance across the drift region, *R*_sub_ is the resistance in the substrate, and *R*_DC_ is the resistance in the drain contact. Therefore, the total ON resistance is the combination of *R*_ch_ and *R*_s_.

30 $$R_{\mathrm{ON}}=\left(\frac{R_{\mathrm{MOS}}}{R_{\mathrm{ch}}}\right)+R_{\mathrm{SC}}+R_{S}+R_{\mathrm{spread}}+R_{\mathrm{drift}}+R_{\mathrm{sub}}+R_{\mathrm{DC}}$$

The device transconductance is considered to extract the value of *R*_s_. By modeling *I*_d_, *g*_m_ can be derived.

31 $$I_{d}=\frac{V_{d}}{R_{\mathrm{ch}}+R_{s}}$$

32 $$g_{m}=\frac{\partial I_{d}}{\partial V_{g}}=\frac{V_{d}}{{\left(R_{s}+R_{\mathrm{ch}}\right)}^{2}}\left(\frac{\partial R_{\mathrm{ch}}}{\partial V_{g}}\right)$$

The accumulation mode MOSFET channel exhibits high electron mobility, and its corresponding MOS resistance in accumulation mode is expressed as

33 $$R_{\mathrm{MOS}}=\frac{L}{t_{c}\mu C_{\mathrm{ox}}\left(V_{g}-I_{d}R_{\mathrm{GS}}-V_{\mathrm{FB}}\right)}$$

where *C*_ox_ represents the gate oxide capacitance, *L* represents the fin length, *t*_c_ represents the fin width, *R*_GS_ denotes the gate-to-source resistance, *V*_FB_ is the flat band voltage, and μ represents the electron mobility. Following the extraction from the transconductance of the device, the electron mobility (μ) is represented as the field effect mobility (μ_FE_). When extracted from the device’s conductance, it is expressed as the effective mobility (μ_eff_) and can be defined as

34 $$\mu_{\mathrm{eff}}=\frac{L}{t_{c}C_{\mathrm{ox}}\left(V_{g}-V_{t}\right)}{\left(\frac{\partial I_{d}}{\partial V_{g}}\right)}_{V_{d}=0}$$

Two key assumptions are made to make parameter extraction easier.

(i) Mobility, μ, depends on gate voltage, *V*_g_.

(ii) At high *V*_g_, the MOS channel is primarily modulated by *V*_g_, whereas the FinFET resistance has minimal dependence on gate voltage bias. The relationship for parallel resistance, incorporating the channel region, is expressed as

35 $$\frac{1}{R_{\mathrm{ch}}}=\frac{1}{R_{\mathrm{fin}}}+\frac{C_{\mathrm{ox}}\mu }{L/t_{c}}\left(V_{g}-I_{d}R_{\mathrm{GS}}-V_{\mathrm{FB}}\right)$$

Based on the two key assumptions, the equation is simplified as

36 $$\frac{\partial \left(\frac{1}{R_{\mathrm{ch}}}\right)}{\partial V_{g}}=\frac{\mu C_{\mathrm{ox}}}{L/t_{c}}=-\frac{1}{R_{\mathrm{ch}}^{2}}\frac{\partial R_{\mathrm{ch}}}{\partial V_{g}}$$

Since *I*_d_ *R*_GS_ ≪ *V*_g_, *I*_d_ *R*_GS_ can be neglected. Substituting eq 41 into eq 32 yields

37 $$g_{m}={\left(\frac{R_{\mathrm{ch}}}{R_{\mathrm{ch}}+R_{s}}\right)}^{2}\frac{\mu_{\mathrm{eff}}C_{\mathrm{ox}}V_{d}}{L/t_{c}}={\left(1-\frac{R_{s}}{R_{\mathrm{ON}}}\right)}^{2}$$

It is rewritten and obtained as

38 $$R_{\mathrm{ON}}=R_{s}-R_{s}\sqrt{\frac{\mu_{\mathrm{FE}}C_{\mathrm{ox}}V_{d}}{L/t_{c}}\frac{1}{\sqrt{g_{m}}}}$$

This model highlights the interdependence of ON resistance with contact resistance, gate control, mobility, and the geometry of the channel. The model is applicable to long-channel vertical FinFETs operating in a quasi-static mode in the moderate-to-strong inversion domain.

## 4 Results and Discussion

The analytical model is validated using TCAD simulations of a field plate-added step-gate GaN FinFET structure. To examine the electrostatic behavior of the structure and channel formation, surface potential is modeled for varying gate biases and presented in Figure 2. In Figure 2a, the off state exhibits a deeper parabolic curve from 1.76 to 0.92 V as the potential barrier is large for zero gate bias, which is shown in Figure 2b. The presence of a potential well indicates the lack of electron accumulation in the channel, resulting in an upward bending of the conduction band (Figure 2c). Further, in Figure 2a, channel formation starts at *V*_g_ > 0.3 V as the potential rises near the source edge to 1.21 V from 0.92 V. The barrier height reduces and falls to 1.51 V as the gate bias rises and gets closer to the threshold voltage (0.6 V), creating a transition from the OFF to ON state. A conductive pathway with a linear surface potential gradient from the source to drain is produced when a large gate bias of *V*_g_ > 0.8 V is applied. This is due to the alignment of the conduction band closer to the Fermi energy level. The potential variation for varying gate bias is substantiated with the electron concentration graph displayed in Figure 2d. The close agreement in analytical and TCAD models in all operating regimes validates the credibility of the developed model.

**Figure 2:**
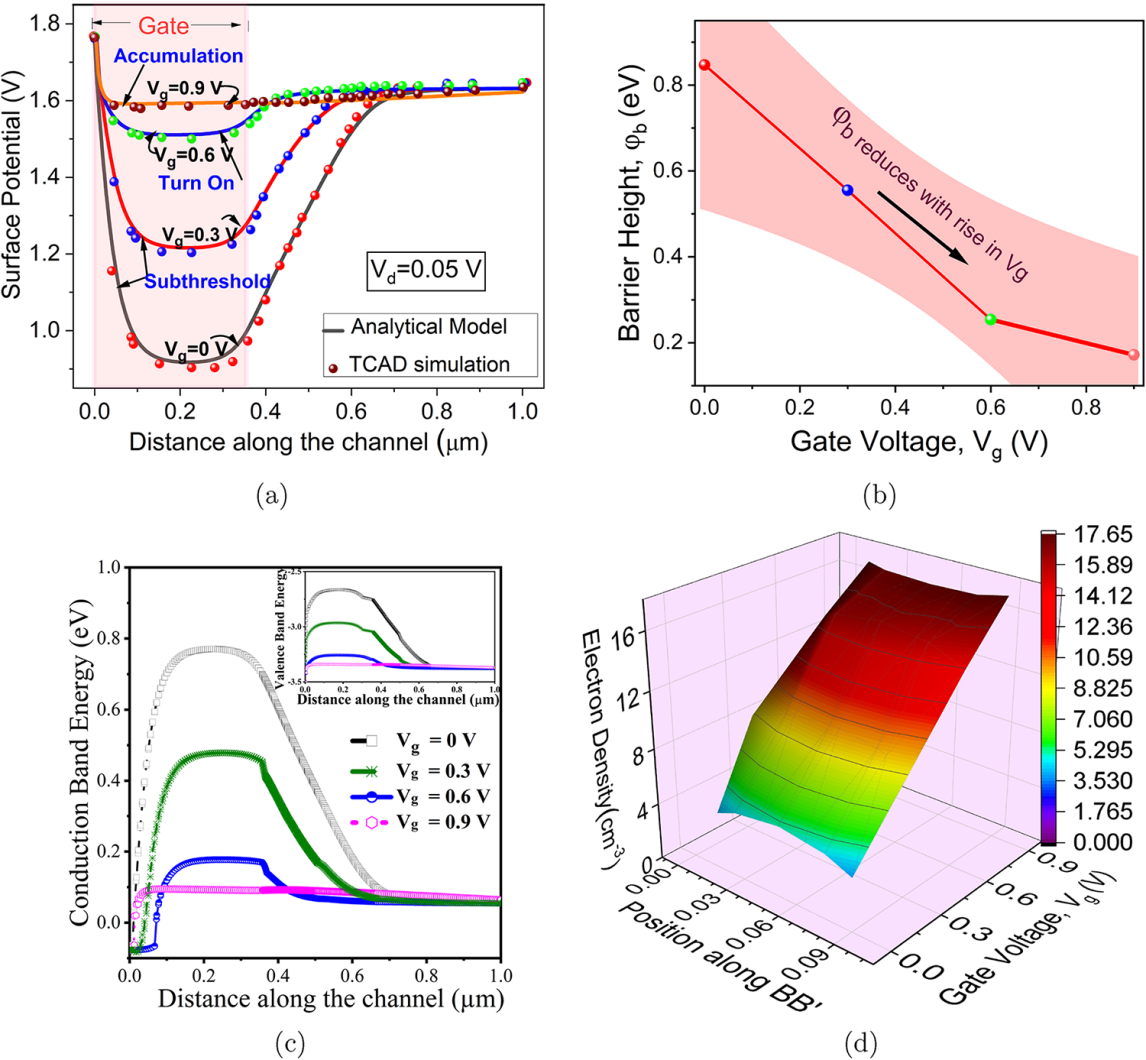
(a) Surface potential, (b) barrier height, (c) conduction band energy, and (d) electron concentration under different gate bias conditions.

The dependency of the doping profile on the surface potential at *V*_g_ = 1 V for the fin channel is demonstrated in Figure 3a. The curve illustrates that a strongly doped channel has a greater surface potential at a fixed gate bias and vice versa (eq 24). For a low-doped channel (4 × 10^15^ cm^–3^), a higher potential barrier is exhibited. As the doping concentration rises to 4 × 10^17^ cm^–3^, the surface potential gradient becomes smoother and uniform, leading to improved conductivity of the device. This is due to the existence of more electrons, resulting in a narrow depletion region and a lower threshold voltage, as the higher carrier density reduces the voltage needed to create an inversion layer. This is substantiated by eqs 12 and 13, where *K*_*i*_^2^ represents the electrostatic coupling and β_*i*_ represents the combined effect of doping and gate bias. A carrier concentration of 4 × 10^19^ cm^–3^ shrinks the depletion width, and the doping term dominates ( $\frac{{\mathrm{qN}}_{R_{i}}}{\varepsilon_{\mathrm{GaN}}}>>K_{i}^{2}V_{g_{i}}$ ), which leads to the gate control and reversal of the potential curve. This also lowers the charge carrier mobility due to impurity scattering. The higher doping also results in the shifting of the threshold voltage to negative, making the device exhibit normal ON behavior. Therefore, it is not recommended to use this doping range.

**Figure 3:**
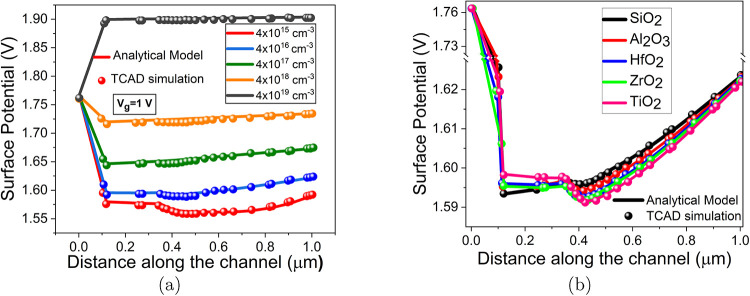
Impact of (a) surface potential under different doping profiles in the fin channel and (b) surface potential plot for different gate oxides.

The surface potential variation for different gate oxides at *V*_g_ = 1 V and *V*_d_ = 50 mV for a fixed gate oxide thickness of 10 nm is illustrated in Figure 3b. Silicon dioxide (SiO_2_) exhibits a moderate modulation of surface potential. The variation in potential across the channel occurs more gradually, necessitating higher gate voltages to achieve significant accumulation. However, the surface potential profiles of high-k gate oxides, such as HfO_2_ (*k* = 20), ZrO_2_ (*k* = 25), and TiO_2_ (*k* = 80), exhibit stable potential and have more control over the channel. The high-*k* oxides support the invasion of EF into the channel, which results in the reduction of surface potential. The carrier concentration affects the turn-on voltage due to the variation of the potential barrier, as shown in Figure 4a. Upon increasing the dopant density, the threshold voltage becomes negative (up to −9.8 V), since the higher carrier density enables the channel to conduct more easily at lower gate voltages.^15^ From the transfer characteristics, the device with a fin width of 100 nm reveals a threshold voltage (*V*_t_) of 0.6 V, at which the device turns ON (Figure 4b). Due to the high dielectric strength of HfO_2_, the leakage current is minimum and has a value in the order of 10^–13^ kA/cm^2^. The cross-sectional area of the fin was used for current normalization, in order to compare with the experimental result,^14^ as it is used for validation.

**Figure 4:**
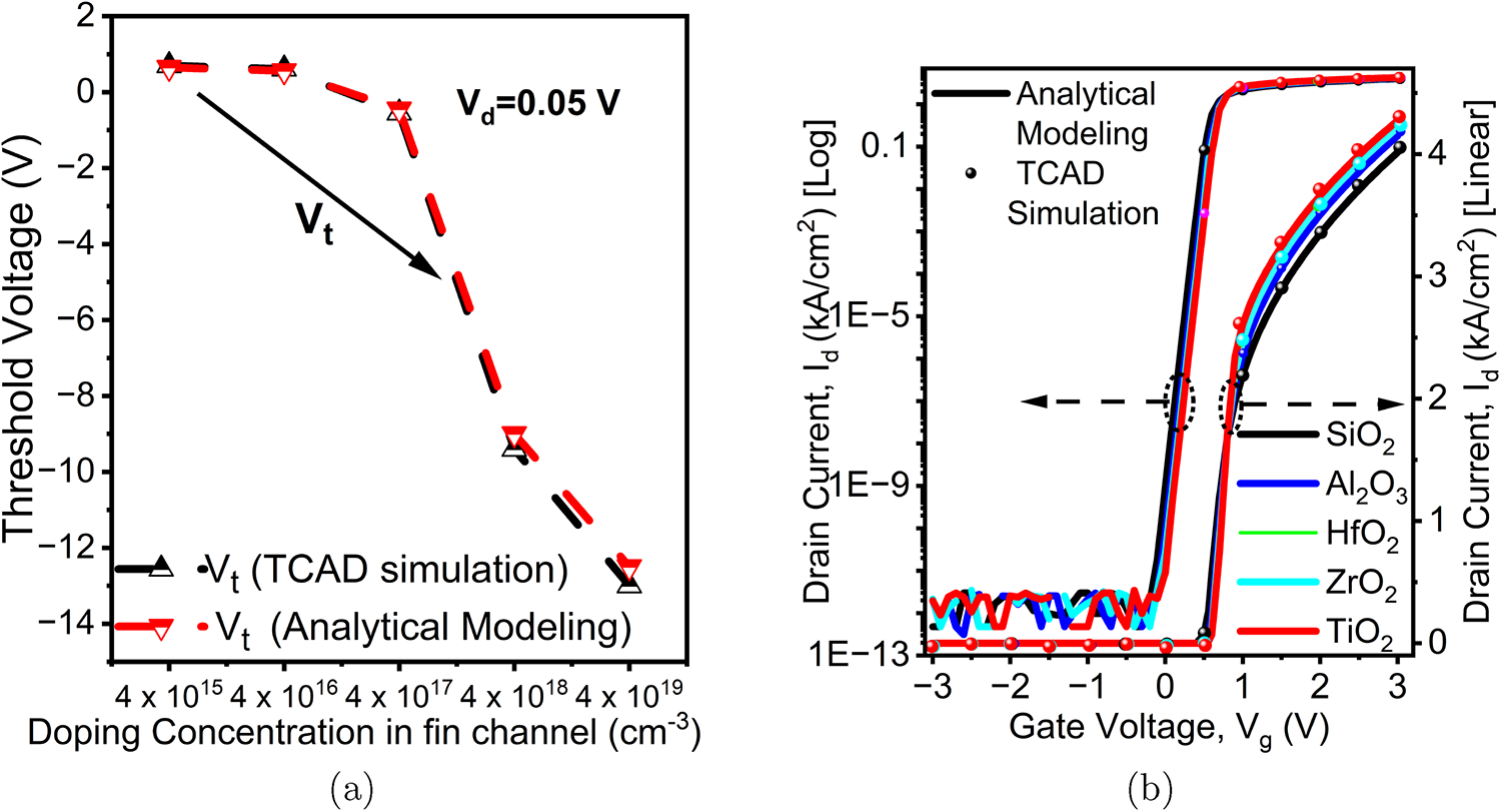
(a) Effect of doping levels on threshold voltage characteristics. (b) Transfer characteristics of a fully vertical GaN FinFET.

The EF is related to the rate of change in surface potential along the channel. This rate of change, or gradient, dictates how quickly charge carriers are accelerated, with a larger gradient leading to a stronger electric field and increased carrier velocity. In Figure 5a, the peak EF is 0.24 MV/cm, observed in the gated channel area. However, the electric field reduces after 0.35 μm along the channel. The vertical electric field (EF) is exhibited in Figure 5b, and the peak EF is denoted as 1.2 MV/cm. As the device is designed with a 350 nm gate length, added with a 500 nm field plate, the device shows a better electric field distribution, and the peak EF is shifted toward the field plate edge. The electric field contour under off-state conditions (*V*_g_ = 0 V and *V*_d_ = 300 V) is displayed in Figure 5c. Under zero gate bias, the EF in the fin channel is weak due to the low surface charge potential, which hinders significant current flow. The electrical field along the channel is characterized by a shallow potential gradient, resulting in minimal conduction. However, at the time of breakdown, the peak field of 2.06 MV/cm can be seen in the edges of the fin, shown in red color. Because of the presence of spacer oxide, the crowding of the electric field near the gate edge and drain side is reduced due to the redistribution of the equipotential lines. Thus, the premature breakdown can be avoided.

**Figure 5:**
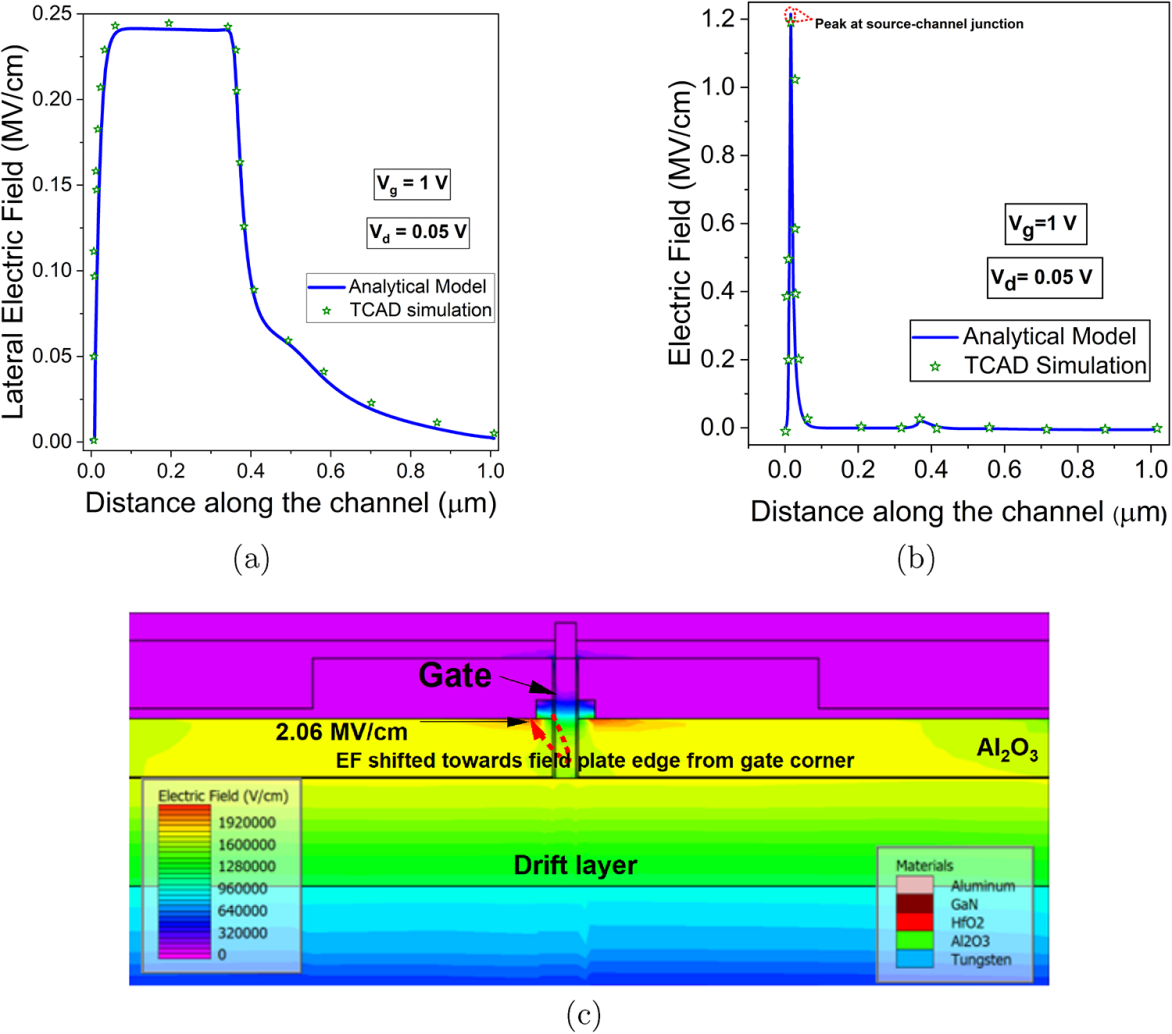
(a) ON state lateral electric field (EF) along the fin channel. (b) ON state vertical electric field (EF) along the fin channel. (c) EF contour at the off state.

The ON resistance in vertical GaN FinFETs is influenced by charge carrier mobility. It consists of multiple components, including resistance from the channel, drift layer, and source/drain access regions, where mobility plays a significant role, as higher mobility eases the carrier transport, thereby reducing resistive losses.^24^ In the GaN fin channel, electron mobility is impacted by factors such as scattering due to the roughness of the surface and quantum confinement due to the fin structure, causing a reduction in mobility compared to that of bulk GaN. The electron mobility along the fin channel at 50 mV drain source voltage is illustrated in Figure 6a. Under unbiased gate conditions (*V*_g_ = 0 V), the mobility of the electron dips around the channel due to the existence of a higher depletion in the channel. As the gate voltage rises around 1–3 V, the accumulation layer is established, and the electron mobility improves to its peak value of 932.8 cm^2^/V s near the fin channel end. However, further increasing the gate voltage leads to stronger carrier confinement, which results in increased scattering effects and leads to a slight decline in mobility. The transconductance (*g*_m_) represents the response in variation of current with respect to the gate voltage variation, expressed in eq 37. At a lower gate bias, the channel formation occurs, leading to an increase in drain current and resulting in a rise in (*g*_m_), as shown in Figure 6b. The transconductance attains a value of 4.44 kS/cm^2^ at *V*_g_ = 0.6 V, where it is at its maximum point. An increase in gate voltage beyond this results in the degradation of the slope of *I*_d_–*V*_g_, leading to a reduction in transconductance after the peak value.

**Figure 6:**
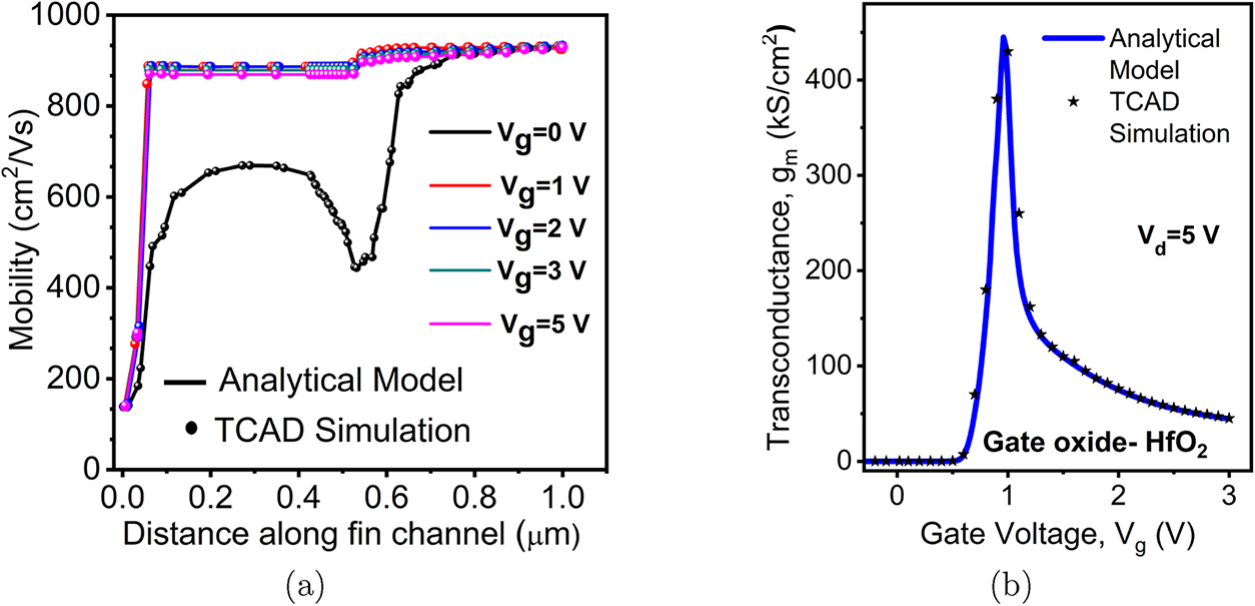
(a) Electron mobility along the channel height. (b) Transconductance characteristics of a fully vertical FinFET.

The operational performance of the device can be analyzed by plotting drain current (*I*_d_) against drain voltage (*V*_d_) for varying gate biases (Figure 7a). The straight-line trend in the triode region demonstrates effective ohmic contact, enabling accurate determination of the channel ON resistance. The *R*_ON,sp_ is evaluated by performing a linear fit for the initial 100 mV of *V*_d_. The normalized ON resistance to the device’s active region is 0.012 mΩ·cm^2^. The lowering of ON resistance is achieved through the optimized concentration of the dopant in the fin-structured channel and the drift layer. The specific contact resistivity extracted from TLM measurements is around 1.5 × 10^–5^ Ω·cm^2^ for the fabricated device, and the analytical model is validated with the experimental data,^14^ showing strong consistency between the data (Figure 7b). Using a 2D device model with *W*_d_ = 3 μm, *N*_d_ = 4 × 10^16^ cm^–3^, and μ = 900 cm^2^/(V s), the drift resistance was estimated as 72 Ω, while the source contact resistance was calculated to be approximately 37 Ω. The overlap between the drain current of the reported data^14^ and the model affirms the validity of the surface potential formulation and its supporting physical assumptions.

**Figure 7:**
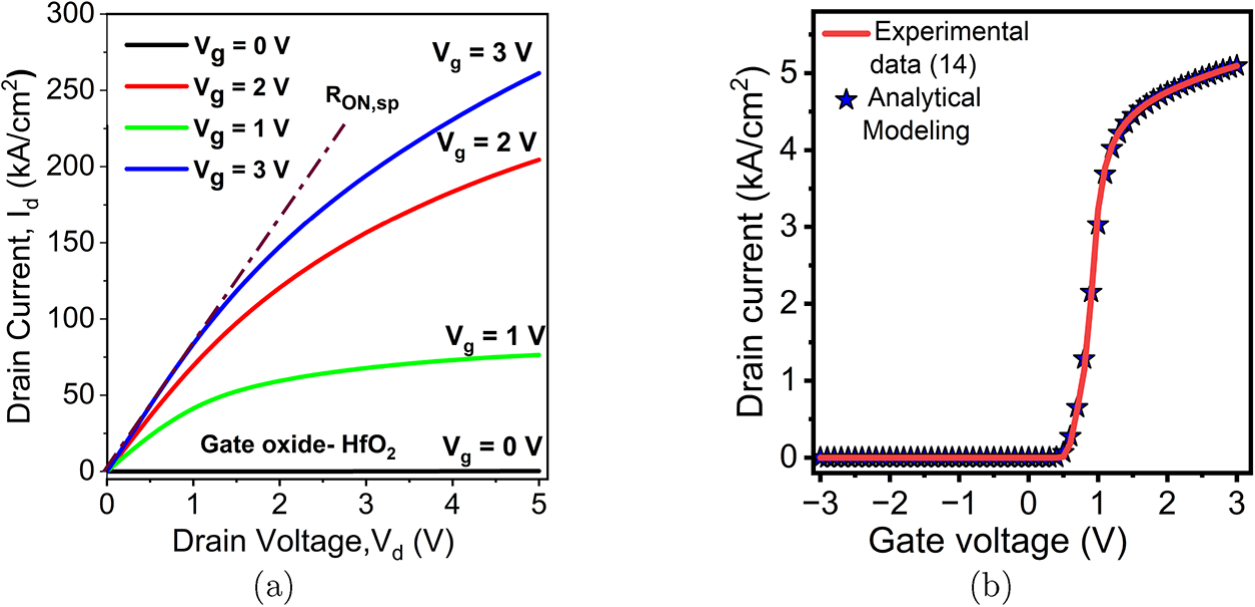
(a) Output characteristics of a stepped gate vertical FinFET. (b) Validation of the analytical model with experimental data.^14^

The validity of the model is applicable for conditions such as moderate channel thickness, uniform material properties, and operation within the electrostatic regime, where higher-order field variations are negligible. The thermal effects are not included in this analytical model. Based on the reported GaN device studies, *R*_ON_ estimation without considering temperature under high-power operation introduces 10–30% deviation.^27^ Therefore, the present model is accurate under moderate bias. The electrothermal modeling of self-heating effects, heat spreading mechanisms, and lattice temperature distribution will be studied in our future work. For further validating the surface potential model in a quantitative way, an error analysis is performed between analytical modeling and TCAD simulation data using the coefficient of determination (*R* ^2^) and root-mean-square error (RMSE). The error comparison has been carried out for surface potential for various gate biases, gate oxides, and fin doping concentrations, as demonstrated in Figure 8a–c. The random forest regressor obtained an *R* ^2^ score of 0.999808 with an RMSE of 0.000643 for surface potential variation with various gate bias conditions. These quantitative analyses produced low RMSE values, and approximately unity *R* ^2^ confirms that the proposed model validates the reliability of the developed analytical framework.

**Figure 8:**
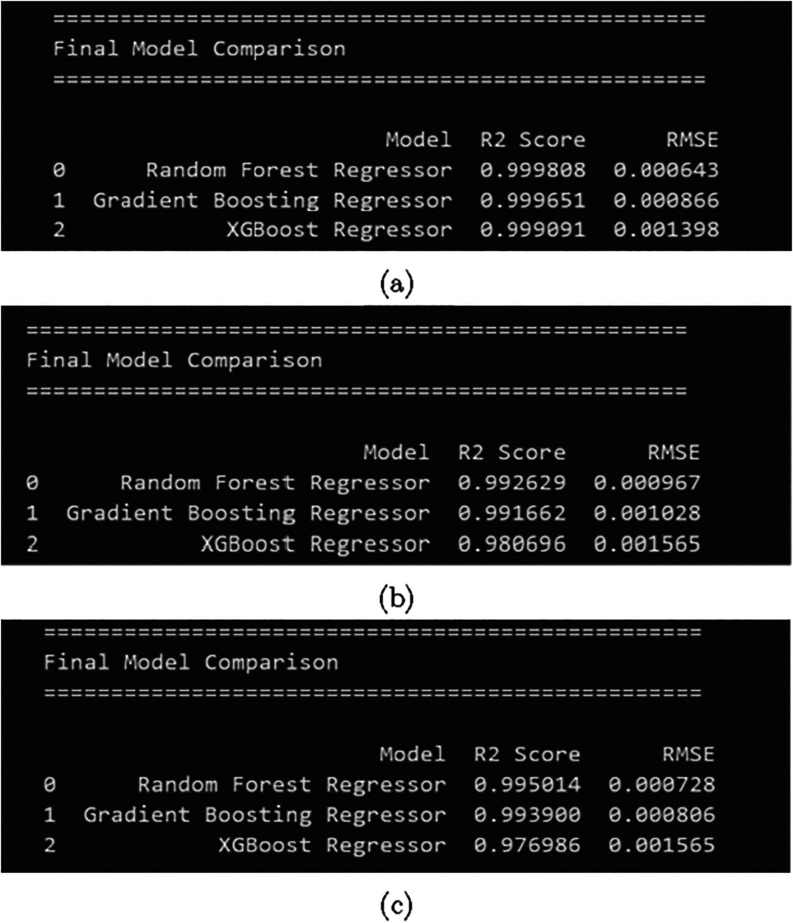
Validation of the analytical model using statistical error analysis. *R* ^2^ score and RMSE for variations in (a) gate bias, (b) gate oxide thickness, and (c) doping concentration.

Table 2 outlines the benchmarking of the proposed model with the existing models. This work considers both gated (L_1_ = *L*_g_) and ungated regions (L_2_ and L_3_) in the fin channel for potential variation, which is not considered in most of the earlier studies. In addition, the present model incorporates gate-field-plate-induced modulation of channel potential in the L_1_ and L_2_ regions, along with coupled gate oxide and spacer oxide electrostatic effects in the L_3_ region. These effects are important for accurately predicting the electric field distribution and improving electrostatic control in vertical GaN FinFET structures. The temperature dependency, line-edge roughness (LER), random dopant fluctuation (RDF), and fringing capacitance are not included in the present work, as the primary focus is on evolving a physics-based electrostatic and current analytical framework for a vertical GaN FinFET structure.

**Table 2:** Benchmark Assessment of the Vertical FinFET

aspects	ref 24	ref 25	ref 26	ref 28	this work
ϕ(*x*) in gated (L_1_) & ungated (L_2_, L_3_) regime	no	yes	yes	no	yes
field plate-induced ϕ(*x*) in L_1_ & L_2_ regimes	no	no	no	no	yes
coupled gate oxide and spacer oxide effects	no	no	no	no	yes
large gate bias	yes	yes	no	yes	yes
large drain bias	no	yes	yes	yes	no
leakage current modeling	no	yes	no	yes	yes
temperature dependent	yes	no	no	no	no
resistance component decomposition	yes	no	no	no	yes
LDR and RDF effects	no	yes	no	no	no
fringing capacitance effects	no	yes	no	no	no

## 5 Device Fabrication Process

The fabrication process steps are explained in Figure 9. First, the GaN layer stack grown by MOVPE is fixed on a GaN substrate (Figure 9a). The next phase is narrow fin patterning, which shapes a vertical fin with steep sidewalls using plasma-reactive ion etching (Figure 9b). The sidewalls are subsequently smoothed by using tetramethylammonium hydroxide (TMAH) wet etching. However, obtaining an exact rectangular region is challenging. The high-*k* gate oxide is deposited by ALD (atomic layer deposition) on both sides of the fin side walls (Figure 9c). Following this is the sputtering of the gate metal combined with the field plate, which is difficult, as the right width and length must be considered (Figure 9d). The top spacer is deposited using ALD to provide a separation between the gate and the source metal (Figure 9e). The conformal deposition of gate oxide and spacer layers over narrow fin structures requires precise ALD process optimization. Finally, the metal contacts (aluminum) for the source and drain are sputtered on both the front and back sides of the developed device (Figure 9f).

**Figure 9:**
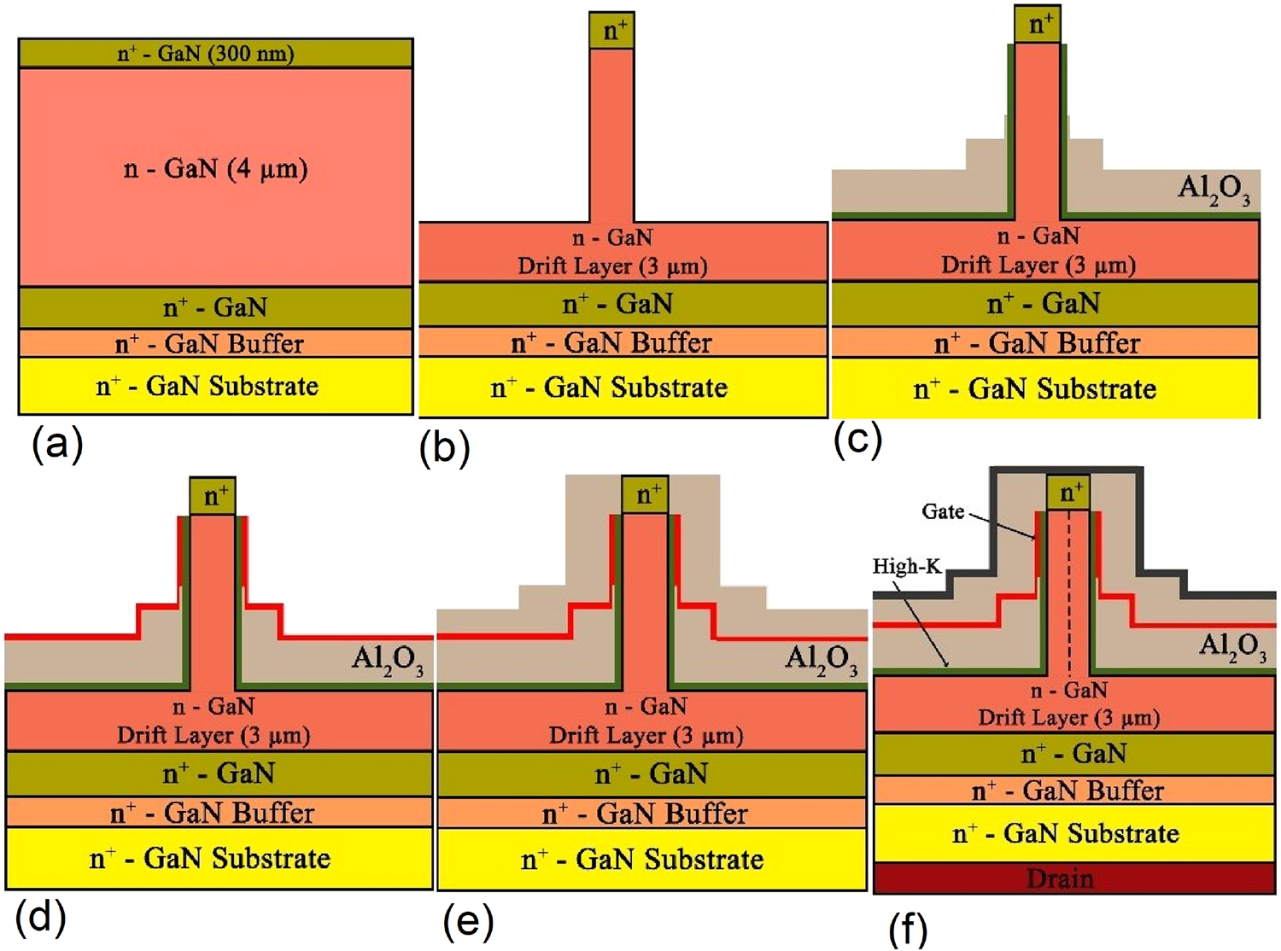
Fabrication process of the stepped gate vertical FinFET: (a) GaN epitaxial layers, (b) fin patterning, (c) gate oxide and spacer oxide formation, (d) gate formation, (e) top spacer deposition, and (f) contact metal deposition.

## 6 Conclusion

In this paper, the surface potential model for the gate field plate-added vertical device is presented, derived from the potential distribution obtained by solving the Poisson equation using a parabolic approximation. The electric field in both lateral and vertical orientations has been derived from the surface potential. The dependence of gate bias, doping concentration, and gate oxide materials on the surface potential is analyzed, highlighting their effects on threshold voltage shift, mobility, and transconductance. The analytical expression for the ON resistance is derived, exhibiting that optimized doping and gate field plate-added structure result in a low *R*_ON,sp_ of 0.012 mΩ·cm^2^ with a 3 μm thick drift layer. This model is validated through comparison with an experimental device and can serve as a useful method for estimating the functionality and operational efficiency of GaN power electronic devices.
